# Successful application of pulsed electromagnetic fields in a patient with post-COVID-19 fatigue: a case report

**DOI:** 10.1007/s10354-021-00901-2

**Published:** 2022-01-10

**Authors:** Barbara Wagner, Margarete Steiner, Lovro Markovic, Richard Crevenna

**Affiliations:** Department of Physical Medicine, Rehabilitation and Occupational Medicine, Medical University of Vienna, Waehringer Guertel 18–20, 1090 Vienna, Austria

**Keywords:** Magnetic field therapy, PEMF, Ion-induction therapy, Long COVID syndrome, Rehabilitation, Magnetfeldtherapie, PEMF, Ioneninduktionstherapie, Long-COVID-Syndrom, Rehabilitation

## Abstract

**Background:**

Post-COVID-19 fatigue is a frequent symptom in COVID-19 survivors, which substantially limits patients to achieve full recovery and potentially restrains return to work. The previous literature has not yet reported the use of pulsed electromagnetic fields in this indication.

**Methods:**

Over the course of 5 weeks, 10 sessions of pulsed electromagnetic field treatment with a high magnetic flux density were applied to a patient suffering from post-COVID-19 fatigue syndrome. Fatigue, work ability, quality of life as well as anxiety, depression, stress level, and resilience were evaluated using validated patient-reported outcome measures.

**Results:**

Fatigue, work ability, quality of life, and psychological well-being improved clearly over the course of the treatment and showed stable results 6 weeks later.

**Conclusion:**

The use of pulsed electromagnetic field therapy with a device that allows sufficient penetration of the body tissue might be a promising physical modality to manage post-COVID-19 fatigue syndrome, which could reduce clinical and economic health consequences. Clinical sham-controlled studies are needed to evaluate the effect of pulsed electromagnetic fields in this indication.

## Abstract (de)

**Grundlagen:**

Das Post-COVID-19-Erschöpfungssyndrom ist ein häufiges Syndrom nach COVID-19, das die vollständige Genesung und möglicherweise auch die Rückkehr in den Arbeitsprozess erheblich einschränkt. In der bisherigen wissenschaftlichen Literatur wurde noch nicht über den Einsatz gepulster Magnetfelder bei dieser Indikation berichtet.

**Methodik:**

Eine Patientin, die an einem Post-COVID-19 Erschöpfungssyndrom litt, wurde über einen Zeitraum von 5 Wochen mit 10 Einheiten gepulster Magnetfeldtherapie von hoher magnetischer Flussdichte behandelt. Müdigkeit, Arbeitsfähigkeit, Lebensqualität, Ängstlichkeit, Depression, Stressniveau und Resilienz wurden mittels validierter Fragebögen erfasst.

**Ergebnisse:**

Die Müdigkeit, Arbeitsfähigkeit, Lebensqualität und das psychische Wohlbefinden besserten sich im Verlauf der Behandlung deutlich und zeigten auch 6 Wochen später stabile Ergebnisse.

**Schlussfolgerungen:**

Die Anwendung der gepulsten Magnetfeldtherapie mit einem Gerät, das eine ausreichende Eindringtiefe in das Körpergewebe ermöglicht, könnte eine vielversprechende physikalische Methode zur Behandlung des Erschöpfungssyndroms nach COVID-19 sein, um die gesundheitlichen und wirtschaftlichen Folgen der Erkrankung zu reduzieren. Klinische placebokontrollierte Studien sind notwendig, um die Wirkung gepulster Magnetfeldtherapie in dieser Indikation zu erforschen.

## Introduction

During the SARS-CoV‑2 pandemic, more than 218.9 million people worldwide have been infected with the virus. In Austria, there have been more than 687,200 confirmed COVID-19 infections; around 676,600 people were considered to be COVID-19 survivors by the beginning of September 2021 [1]. Post-COVID-19 fatigue syndrome is primarily associated with physical weakness, tiredness, and exhaustion. Fatigue affects roughly 85% of patients hospitalized due to COVID-19 [2] and over 60% of patients at 5–6 months from symptom onset [3]. Thus, it substantially limits patients ability to achieve full recovery and potentially restrains return to work. Further common physical and mental health sequelae of post-COVID-19 are musculoskeletal pain, reduced physical capacity, anxiety, depression, posttraumatic stress disorder, and overall lower quality of life [4]. There is a need for further research on effective rehabilitation strategies to manage these conditions [3].

Electromagnetic fields can affect biological structures such as body cells and tissues and can induce selective changes in their microenvironment [5]. The effect is athermal [5, 6]. Exposing the body to electromagnetic fields is possible through either capacitive coupling (placing opposite electrodes within a conducting medium) or through inductive coupling (a time-varying pulsed electric field induces an electric current in the target tissue) [5].

To our knowledge, this is the first scientific report in which pulsed electromagnetic field (PEMF) treatment has been applied in the rehabilitation of a patient suffering from post-COVID-19 fatigue syndrome.

## Case report

A 55-year-old female presented with persisting weakness, tiredness, and exhaustion since the infection with SARS-CoV‑2 6.5 months ago. COVID-19 disease had initially caused respiratory symptoms, myalgia, cephalea, and anosmia. She had been on sick leave for 3 weeks, taking mefenamic acid (Parkemed® 500 mg) twice daily for pain control. At her initial visit to our outpatient clinic, her main complaint was fatigue, which severely affected her workability, along with difficulty in concentrating and persisting exertional dyspnea. Relevant pre-existing conditions were a nodular euthyroid goiter, restless legs syndrome, and climacteric complaints (excessive perspiration). Except for an estrogen drug (17-beta-estradiol, Estrogel Gel® 80 g) that the gynecologist had prescribed for menopausal symptoms, she took no medication. She was married, had two children, and worked 30 h a week. Complaints had been aggravated due to heavy mental workload after the sick leave. Another stress factor were periods of homeschooling for her children due to three lockdowns over much of the time until 2 months before PEMF treatment started.

Her height, weight, and body mass index were 177 cm, 70 kg, and 22 kg/m^2^, respectively. Auscultation of the heart and lungs was normal. Blood pressure was 130/80 mm Hg. Blood values showed a normal hemogram except for minimally decremented leucocytes (3.69 G/L), and normal CRP, electrolytes, 25-OH vitamin D, iron, liver, and kidney values as well as thyroidal and metabolic parameters. Pulmonologist checkup turned out normal as well.

The patient had not received any treatment for her fatigue symptoms specifically. She had started supervised resistance training for 10 different muscle groups twice weekly 5 months previously. The training involved two sets of 13–15 repetitions and was progressed as strength improved. Subjectively, the training had not changed fatigue symptoms.

### Methods

The patient gave informed consent to receive PEMF to treat fatigue symptoms and to document the findings for a case study. We used the Papimi™ electromagnetic field therapy device (Pulse Dynamics Ltd., Pipinou 1 & Souliou, 17342 Agios Dimitrios, Greece), which is a certified and approved medical device (class IIa) that can be safely and effectively applied to treat fatigue and general weakness [6, 7]. It is based on the principle of ion induction. The Papimi™ pulse is like a damped oscillation with a short pulse duration of ~ 50 µs. The basic frequency is ~ 240 kHz; in the maxima and minima of the damped oscillation, high-frequency oscillation peaks in the megahertz to gigahertz range arise. The pulse rate can be varied between 1 and 8 Hz. High voltages (up to 40 kV) and peak currents (up to 10 kA) arise in the applicator spool. As a result, the Papimi™ device achieves delivery of energy per pulse of about 96 Ws (Joule) with a magnetic flux density of 50–100 mT [6].

Contraindications to PEMF therapy, which include electronic implants (e.g., pacemaker, implantable cardioverter defibrillator, cochlear implants), pregnancy, and ring-shaped metals in the body [6], were ruled out prior to starting the treatment.

The patient received 10 sessions of PEMF twice weekly for 5 weeks, each session lasting 30 min. The treatment protocol was chosen as follows: starting with the patient supine, 6 min in the epigastric/abdominal area, and 3 min over the sternum were administered. Then, 6 min were applied in the dorsal area (covering the lungs and the adrenal area), 6 min in the pelvic floor area, and 6 min on the soles of both feet. Locations of application were chosen following the device manufacturer’s manual [8]. The pulse rate was 2.5 Hz in the dorsal area and 1 Hz for all other locations. Treatment intensity was adapted as tolerated, choosing the distance between the applicator spool and the patient’s skin between 0 and 4 cm. Initially, the smaller treatment spool (diameter 18 cm) was used, during the course of the treatment the larger spool (diameter 20 cm) was applied for all areas except the pelvic floor. Most of the time, 75% of maximum power was chosen for the abdominal, sternal, and pelvic floor area and 100% for the dorsal and plantar applications.

Since long COVID-19 syndrome is associated with both physical and mental health issues [4], the following validated patient-reported outcome tools were chosen to evaluate the treatment effect pre- and post-intervention (Table 1): The Brief Fatigue Inventory (BFI) measures severity and impairment from fatigue [9]. The Short Form (SF-36) Health Survey is a questionnaire to measure health-related quality of life [10]. The Work Ability Index (WAI) short version evaluates work ability in relation to current physical and psychological work demands [11, 12]. The Perceived Stress Scale (PSS-10) assesses the subjective experience of stress, including the subscales helplessness and self-efficacy [13]. The Patient Health Questionnaire 9 (PHQ-9) is a tool to screen for the presence and severity of depression [14]. The Generalized Anxiety Disorder 7 (GAD-7) is a questionnaire to identify patients with a generalized anxiety disorder and capture the severity of symptoms [15, 16]. The Brief Resilience Scale (BRS) measures the ability to successfully cope with difficult, stressful situations and to recover quickly from them [17, 18].

**Table 1:** Questionnaire results pretreatment, posttreatment, and 6 weeks after treatment

	Subscales	Range (points)	Score interpretation	Results (see legend)		
				T1	T2	T3
BFI	**BFI total score**	[0–10]	*Fatigue:* 1–3: mild 4–6: moderate 7–10: severe	**6.33**	**0.22**	**0.11**
	Fatigue right now			7	0	0
	Usual fatigue in last 24 h			7	1	0
	Worst fatigue in last 24 h			7	1	1
	General activity			3	0	0
	Mood			8	0	0
	Walking ability			0	0	0
	Normal work (incl. housework)			7	0	0
	Relations with other people			9	0	0
	Enjoyment of life			9	0	0
SF-36	Physical functioning	[0–100]	*Quality of life:* 0: worst value 100: best value	60	90	90
	Role physical			25	100	100
	Role emotional			0	100	100
	Vitality			10	85	95
	Mental health			44	96	92
	Social functioning			25	87.5	100
	Bodily pain			41	84	84
	General health perceptions			55	100	100
WAI	**WAI (short version)**	[7–49]	*Work ability:* 7–27: critical 28–36: moderate 37–43: good 44–49: very good	**21.5**	**40**	**40**
PSS-10	**PSS-10 total score**	[0–50]	*Stress level (total score):* 0–13: low 14–26: moderate ≥ 27: high	**43**	**16**	**16**
	Helplessness	[6–30]		27	11	10
	Self-efficacy	[4–20]		8	19	18
PHQ‑9	**PHQ‑9**	[0–27]	*Depression:* 5–9: mild 10–14: moderate 15–19: moderately severe 20–27: severe	**16**	**2**	**1**
GAD‑7	**GAD‑7**	[0–21]	*Generalized anxiety:* 5–9: mild 10–14: moderate 15–21: severe	**13**	**2**	**2**
BRS	**BRS**	[1–5]	*Resilience:* 1.00–2.99: low 3.00–4.30: normal 4.31–5.00: high	**2.67**	**4.67**	**4.67**

*BFI* Brief Fatigue Inventory [9], *BRS* Brief Resilience Scale [17, 18], *GAD‑7* Generalized Anxiety Disorder 7 [15, 16], *PHQ‑9* Patient Health Questionnaire 9 [14], *PSS-10* Perceived Stress Scale [13], *SF-36* Short Form 36 Health Survey [10], *WAI* Work Ability Index [11, 12], *T1* before treatment, *T2* immediately after treatment, *T3* follow up 6 weeks after treatment. The German versions of the respective questionnaires were used

### Results

The patient reported an increase in energy and a decrease of fatigue symptoms as of the fourth treatment session, with improvements continuing over the following sessions. After completing all 10 sessions, she felt fully recovered and able to face challenges in work and private life. Wellbeing continued at the follow-up evaluation 6 weeks later. No significant side effects had occurred apart from an increase in neck pain during local PEMF application, which disappeared after adapting treatment intensity in this specific area, and a short-term increase in fatigue after the first session.

The results of all questionnaires that were administered before (T1), immediately after (T2), and 6 weeks (T3) after the end of the treatment reflected the patient-reported outcome (Table 1). The BFI score decreased from 6.33 (moderate fatigue) at baseline to 0.22 (no fatigue) post intervention. The largest improvements were seen in the dimensions of mood, work, relationship, and enjoyment of life. The SF-36 also showed an increase in several dimensions. Workability improved from critical (21.5 points) before treatment to good (40 points) after the intervention. The pre-intervention total score of the PSS-10 decreased from a high (43 points) to a moderate (16 points) stress level post intervention. The value for the subscale helplessness decreased from 27 to 11 points and, on the other hand, the value for self-efficacy increased from 8 to 19 points. The PHQ‑9 score improved from 16 points (indicating moderately severe depression) to a normal level (2 points). The GAD‑7 score also decreased from a pathologic (13 points) to a normal value (2 points) during the treatment. Resilience level improved from low (2.67 points) to high (4.67 points) after treatment. Improvements persisted in all areas, as the follow-up evaluation showed 6 weeks later. To give us examples of her new energy levels, the patient reported that 3‑h hikes or baking a cake at the end of a busy day were no longer a problem.

## Discussion

In patients suffering from fatigue, PEMF treatment has been applied primarily in patients with multiple sclerosis, with mixed success [19–27].

Similarly, randomized controlled trials (RCTs) on magnetic therapy in fibromyalgia patients showed mixed results: in one trial, PEMF mat therapy improved pain, fibromyalgia impact questionnaire, and quality of life compared to placebo [28], whereas another study showed no superiority of PEMF over placebo in improving pain and function [29]. In chronic musculoskeletal pain (including fibromyalgia patients), PEMF had no significantly differential advantage over sham treatment [30]. Regarding static magnetic devices, mattress pads during sleep provided statistically significant pain relief and sleep improvement [31], whereas another trial found low static magnetic fields only superior to sham/usual care in terms of decreased pain levels [32]. Repetitive transcranial magnetic brain stimulation relieved pain and enhanced quality of life in patients with fibromyalgia but did not improve other symptoms, as shown in a recent systematic review [33].

In workers with work-related chronic stress, an RCT found no additional effect of light plus PEMF therapy on return to work, fatigue, stress, and quality of life compared to coaching alone [34].

However, low-frequency PEMF therapy has already been used successfully in a Russian study in the rehabilitation of 52 patients after COVID-19 pneumonia. PEMF decreased respiratory symptoms, pain, anxiety, and depression, and improved quality of life [35].

The particular physical parameters of the applied electromagnetic fields appear to have a significant impact on the observed effect on biologic structures. Devices with short pulse duration and a wide frequency spectrum may provide a higher chance of activating cellular reactions [5]. The particular physical parameters of electromagnetic devices have to be considered when studies are compared and results are interpreted [5]. Unfortunately, necessary details characterizing an electromagnetic device such as the type of the field, the intensity of the induction, frequency, pulse rate of rise and decline, pulse shape, and vector or time of exposure are rare information even in scientific publications and vary greatly among different treatment protocols. Therefore, comparisons between existing studies and qualified ratings are often difficult [5]. Many of the previous studies on multiple sclerosis-associated fatigue [20–27] and on fibromyalgia [28–30, 32] were performed with magnetic flux densities in the microtesla range, using very heterogeneous treatment parameters. Therefore, these protocols are hardly comparable to the one used in this case.

This single case study showed promising results upon applying PEMF in a patient suffering from post-COVID-19 fatigue. The literature reports associations between physical symptoms and mental health issues such as anxiety and depression [4]. Our results can similarly be interpreted to the effect that an improvement in the physical energy level appears to have a clear influence on psychological wellbeing and resilience.

The exact mode of action of electromagnetic fields is unknown [5]. Among other hypotheses, it is suggested that electromagnetic stimuli interact with cells via either ion channels or transmembrane receptors, thereby initiating signal transduction cascades or modifying cellular functions [5, 36]. Brief exposure to low millitesla-range PEMF was recently shown to enhance mitochondriogenesis [37] and modulate metabolism and gut microbiome [38] in a cell study and animal experiment. The gut microbiome–immune system–brain axis is, in turn, sensitive to stress and plays an important role in the development of stress-related symptoms such as a major depressive disorder [39], which might explain the benefit of the PEMF application on certain psychological parameters.

A limitation of this case report could be that factors other than PEMF may have contributed to an improvement in symptoms. Physical exercise, particularly aerobic plus resistance training, can improve cancer-related fatigue [40]. However, the patient started physical exercise 5 months before PEMF therapy and had not seen any subjective improvement on fatigue until then. Up to 2 months before the start of PEMF treatment there was homeschooling. Psychological factors such as her decision to enhance self-care and reduce weekly working hours in the future may also have played a role. However, it can be assumed that an improvement of the individual parameters to this extent over a treatment period of only 5 weeks would not have been expected without intervention, considering that the complaints had already persisted for 6.5 months.

Magnetic field therapy based on ion induction therapy is well tolerated, noninvasive, and easily applied to the fully dressed patient [5]. According to manufacturer specifications of the Papimi™ device, dizziness, fatigue, nausea, or headache (in 1.5% of those treated) may occur after the first treatment as well as a short-term worsening of the initial symptoms [6]. The literature does not report any significant side effects from PEMF treatment [36, 41].

Since this application of PEMF in post-COVID-19 fatigue syndrome showed promising results, we aim to evaluate the effect of PEMF on both patient-reported and physical performance outcomes in a larger patient sample. If the results are appropriate, magnetic field therapy could be an important treatment option in addition to physical exercise in the rehabilitation of patients with post-COVID-19 fatigue syndrome. It may also serve as a treatment strategy on its own in patients with contraindications to physical exercise or significant limitations due to underlying cardiopulmonary or orthopedic diseases.

## Conclusion

Post-COVID-19 fatigue is a frequent symptom that currently affects many COVID-19 survivors. PEMF is well tolerated, easy to apply, and has shown promising results in this case study. The use of PEMF therapy with a device that allows sufficient penetration of the body tissue might be a promising physical modality to manage post-COVID-19 fatigue and associated symptoms, which could reduce economic and clinical health consequences. Clinical sham-controlled studies are needed to evaluate the effect of PEMF on patients with post-COVID-19 fatigue syndrome. Attention should be paid to an exact specification of the physical characteristics of the respective PEMF device and the description of the treatment protocol, so that studies are comparable and study data can be interpreted correctly.
